# Tandem Mass Spectrometry for ^13^C Metabolic Flux Analysis: Methods and Algorithms Based on EMU Framework

**DOI:** 10.3389/fmicb.2019.00031

**Published:** 2019-01-24

**Authors:** Jungik Choi, Maciek R. Antoniewicz

**Affiliations:** Department of Chemical and Biomolecular Engineering, Metabolic Engineering and Systems Biology Laboratory, University of Delaware, Newark, DE, United States

**Keywords:** elementary metabolite units, metabolic flux analysis, tandem mass spectrometry, stable isotope tracers, metabolism

## Abstract

In the past two decades, ^13^C metabolic flux analysis (^13^C-MFA) has matured into a powerful and widely used scientific tool in metabolic engineering and systems biology. Traditionally, metabolic fluxes have been determined from measurements of isotopic labeling by means of mass spectrometry (MS) or nuclear magnetic resonance (NMR). In recent years, tandem MS has emerged as a new analytical technique that can provide additional information for high-resolution quantification of metabolic fluxes in complex biological systems. In this paper, we present recent advances in methods and algorithms for incorporating tandem MS measurements into existing ^13^C-MFA approaches that are based on the elementary metabolite units (EMU) framework. Specifically, efficient EMU-based algorithms are presented for simulating tandem MS data, tracing isotopic labeling in biochemical network models and for correcting tandem MS data for natural isotope abundances.

## Introduction

^13^C-Metabolic flux analysis (^13^C-MFA) is a widely used technique in metabolic engineering and biomedical sciences for quantifying rates of metabolite interconversions inside living cells (i.e., *in vivo* metabolic fluxes) (Antoniewicz, 2015a,b; Ahn et al., 2016). In ^13^C-MFA, labeling experiment are performed by introducing a ^13^C labeled substrate (the tracer), followed by measurement of ^13^C labeling incorporation, and calculation of metabolic fluxes using one of several available software packages for ^13^C-MFA (Yoo et al., 2008; Quek et al., 2009; Weitzel et al., 2013; Young, 2014). Currently, the main techniques used to measure ^13^C labeling are mass spectrometry (MS) (Antoniewicz et al., 2007a, 2011; McConnell and Antoniewicz, 2016), nuclear magnetic resonance (NMR) spectroscopy (Masakapalli et al., 2014), and tandem MS (Antoniewicz, 2013; Okahashi et al., 2016). Previous studies have demonstrated that tandem MS measurements can provide more labeling information than MS resulting in improved performance of ^13^C-MFA in complex biological systems (Jeffrey et al., 2002; Choi and Antoniewicz, 2011; Choi et al., 2012).

Various modeling approaches have been applied to simulate tandem MS data for applications in ^13^C-MFA. These include manually derived algebraic equations for specific network models (Jeffrey et al., 2002), isotopomers (Choi and Antoniewicz, 2011; Chandra and Peng, 2012), and tandemers (Tepper and Shlomi, 2015). Despite the demonstrated advantages of tandem MS, the technique is still not fully embraced by the ^13^C-MFA community, likely because the modeling approaches have not been based on the elementary metabolite units (EMU) framework (Antoniewicz et al., 2007b; Crown and Antoniewicz, 2012), which is the most widely used approach for modeling isotopic labeling in ^13^C-MFA (Young et al., 2008). The EMU framework is at the core of all major software packages for ^13^C-MFA (Yoo et al., 2008; Quek et al., 2009; Weitzel et al., 2013; Young, 2014). In this methods paper, we describe efficient algorithms for modeling tandem MS data that are firmly based on the EMU framework. By building upon the EMU framework, we illustrate that the presented algorithms can be easily incorporated into existing software packages to take full advantage of the additional information provided by tandem MS for high-resolution flux measurements.

## Simulation of Tandem MS Data

### Compact Tandem MS Matrix

First, we introduce here the new concept of the compact tandem MS matrix that will be used throughout this paper to describe tandem MS data. In later sections, it will be demonstrated that the compact tandem MS matrix is compatible with the EMU framework to allow tandem MS data to be incorporated into existing EMU algorithms for ^13^C-MFA.

**FIGURE 1:**
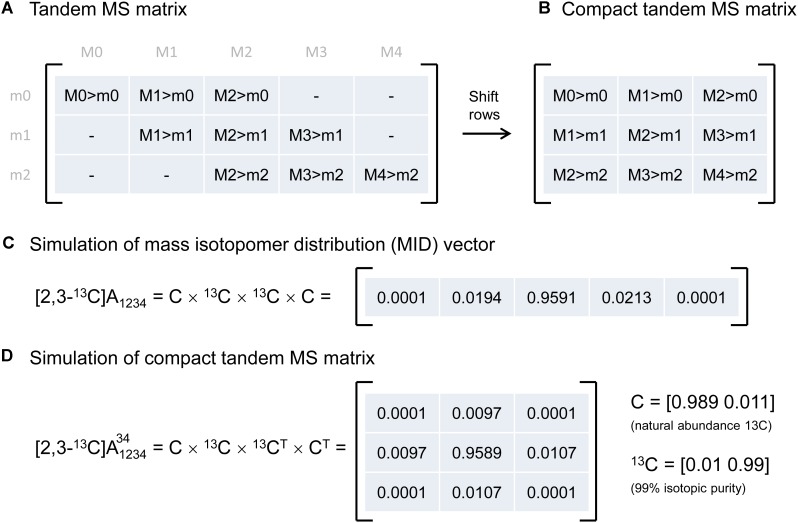
**(A)** Representation of tandem mass spectrometry data using a tandem MS matrix, and **(B)** a compact tandem MS matrix. **(C)** A mass isotopomer distribution vector can be simulated efficiently by a series of convolutions (denoted by “×”). **(D)** A compact tandem MS matrix can be simulated analogously by a series of 2D-convolutions (denoted by “×”). For illustration purposes, the MID vector and the compact tandem MS matrix were simulated for a four-carbon metabolite A that is ^13^C-labeled at carbon positions 2 and 3.

Tandem MS data was previously represented by the so-called tandem MS matrix (Figure 1A; Choi and Antoniewicz, 2011), where the columns correspond to *m/z* of the parent fragment and rows correspond to *m/z* of the daughter fragment. As an example, consider a metabolite A that has four carbon atoms, and assume that carbon atoms C1–C4 are present in the parent fragment, and that carbon atoms C3–C4 are present in the daughter fragment. In this case, the tandem MS matrix is a 3 × 5 matrix (rows m0–m2, columns M0–M4; Figure 1A). A disadvantage of this representation is that several matrix fields will have zero values by definition. For example, in the first column of the tandem MS matrix, which corresponds to the unlabeled parent fragment (M0), the only possible daughter fragment is unlabeled (m0). A more convenient way of representing tandem MS data is by defining the compact tandem MS matrix, which is constructed by shifting rows of the tandem MS matrix to eliminate fields that are infeasible (i.e., which are zero by definition). In this example, the compact tandem MS matrix is 3 × 3 matrix (Figure 1B).

### Simulating Tandem MS Data Using 2D-Convolutions

Next, we describe an algorithm to simulate the compact tandem MS matrix for metabolites that are naturally labeled and for isotopic tracers that are labeled at specific carbon positions. Previously, we demonstrated that mass isotopomer distributions (MID) can be simulated efficiently using a series of convolutions (function *conv* in Matlab) (Antoniewicz et al., 2007b), essentially by reconstructing a metabolite atom-by-atom (Figure 1C). Here, we demonstrate that the compact tandem MS matrix can be simulated analogously using a series of 2D-convolutions (function *conv2* in Matlab), again by reconstructing a metabolite atom-by-atom. This approach can be used to simulate both naturally labeled compounds and tracers that are labeled at one or more specific carbon positions.

As an example, assume that we want to simulate the compact tandem MS matrix for metabolite A that is labeled at the second and third carbon positions, i.e., [2,3-^13^C]A, and assume that the parent fragment contains all four carbon atoms, and the daughter fragment contains carbon atoms 3 and 4. Simulation of the compact tandem MS matrix is achieved by a sequence of 2D-convolutions as shown in Figure 1D. The 2D-convolution is performed with the transpose of the atom’s MID vector if the atom is present in the daughter fragment, and using the atom’s MID vector if the atom is not present in the daughter fragment. The simulated compact tandem MS matrix for [2,3-^13^C]A is shown in Figure 1D. The same approach can be used to simulate compact tandem MS matrices for metabolites of any size. This approach is not limited to simulating only carbon atoms, but can also be used to include any and all atoms. This is important since in LC-MS/MS natural abundances of e.g., sulfur (4.2% M+2) and oxygen (0.2% M+2) contribute to shifts in tandem MS distributions, and even more importantly, in GC-MS/MS analysis compounds are often derivatized (e.g., with TBDMS) which adds other atoms to the fragment that cause even more dramatic shifts in tandem MS distributions (Choi et al., 2012; Okahashi et al., 2016).

## Correction of Tandem Ms Data

### Parent, Daughter, and Complement Fragments

**FIGURE 2:**
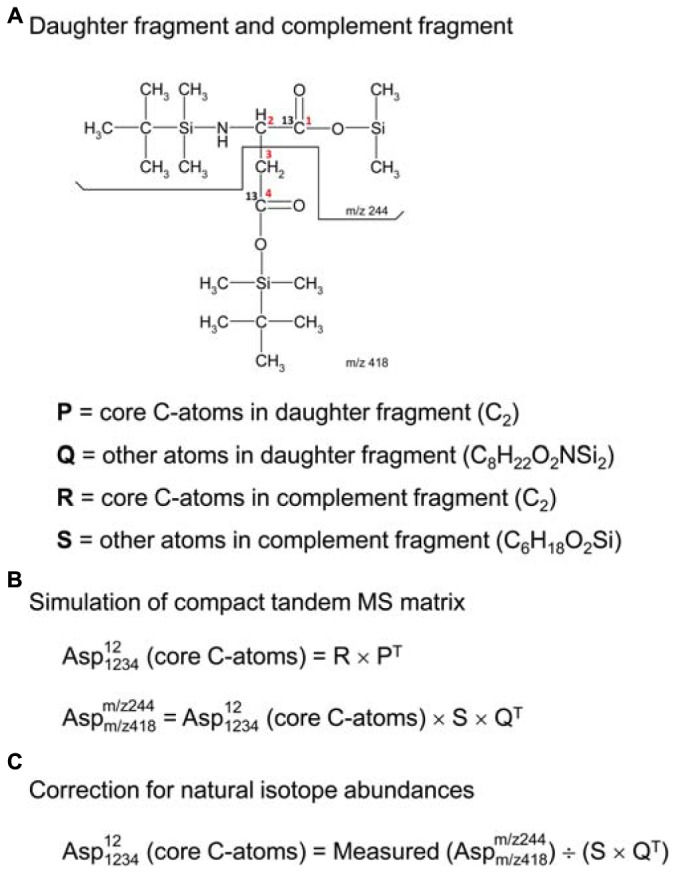
**(A)** Definitions of daughter fragment and complement fragment; here, illustrated for TBDMS-derivatized aspartate, where the daughter fragment contains carbon atoms C1 and C2 of aspartate, and the complement fragment contains carbon atoms C3 and C4 of aspartate. **(B)** The compact tandem MS matrix of TBDMS-derivatized aspartate is simulated using 2D-convolutions (denoted by “×”), where Q and S are the natural abundance MID vectors for the other atoms (i.e., non-core C-atoms) of the daughter and complement fragments, respectively. **(C)** Correction for natural isotope abundances of non-core C-atoms is accomplished by a 2D-deconvolution (denoted here by “÷”).

In order to use tandem MS data for ^13^C-flux calculations, the data must be corrected for natural isotope abundances. Efficient algorithms have been developed for correcting MS data for use in ^13^C-MFA (Fernandez et al., 1996); however, existing algorithms for correcting tandem MS data tend to be more tedious (Rantanen et al., 2002; Niedenfuhr et al., 2016). Here, we describe a more convenient algorithm based on the compact tandem MS matrix formulation described above. First, we must define a new term, the “complement fragment,” shown in Figure 2A. The complement fragment is defined as the part of the parent fragment that is not present in the daughter fragment. The daughter fragment and the complement fragment can then be further imagined to consist of core carbon atoms that originate from the metabolite that is measured, and other atoms (which may include both carbon and non-carbon atoms) from the derivatizing agent. For ^13^C-MFA calculations, we are only interested in the labeling of the core C-atoms; thus, tandem MS data must be corrected for the skewing effects resulting from the presence of carbon atoms that are not core C-atoms.

### Correcting Tandem MS Data for Natural Abundances

The correction of tandem MS data for natural isotope abundances is accomplished using 2D-deconvolution. To illustrate this, consider the case of TBDMS-derivatized aspartate. In a previous study, we validated several parent-daughter GC-MS/MS fragments for analysis of aspartate labeling (Choi et al., 2012). One of the validated parent-daughter pairs was *m/z* 418 > 244 (Figure 2A). In this case, the parent fragment (*m/z* 418, C_18_H_40_O_4_NSi_3_) contains all four C-atoms of aspartate and the daughter fragment (*m/z* 244, C_10_H_22_O_2_NSi_2_) contains the first two C-atoms of aspartate. Following the definitions in the previous section, the daughter fragment is thus imagined to consist of two core C-atoms of aspartate (i.e., C_2_, first two carbon atoms), and various other atoms (C_8_H_22_O_2_NSi_2_); the complement fragment consists of two core C-atoms of aspartate (i.e., C_2_, last two carbon atoms), and various other atoms (C_6_H_18_O_2_Si). If the labeling of the core C-atoms is known, then we can predict the theoretical measured compact tandem MS matrix by 2D-convolutions shown in Figure 2B, where S is the natural abundance MID vector of C_6_H_18_O_2_Si, and Q is the natural abundance MID vector of C_8_H_22_O_2_NSi_2_. The inverse operation, or 2D-deconvolution, can therefore be used to correct the measured tandem MS data for natural isotope abundances (Figure 2C). It should be noted that 2D-deconvolutions of this type are widely used in many fields of science such as image processing and filtering (e.g., functions *fft2* and *ifft2* in Matlab).

**FIGURE 3:**
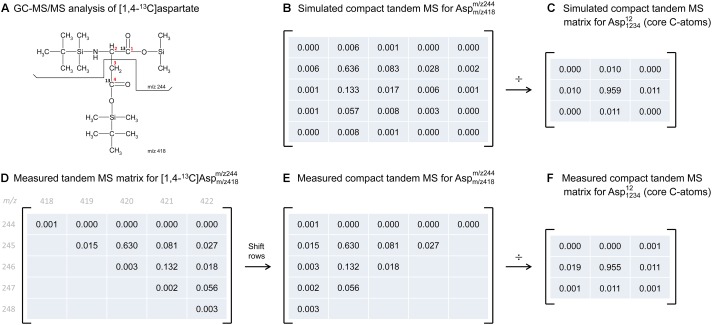
Illustration of natural abundance correction for both simulated and measured tandem MS data. **(A)** GC-MS/MS analysis was performed on TBDMS-derivatized [1,4-^13^C]aspartate, where the parent fragment contains all four carbon atoms of aspartate, and the daughter fragment contains carbon atoms C1 and C2 of aspartate. **(B)** The predicted compact tandem MS matrix for [1,4-^13^C]aspartate *m/z* 418 > 244 parent-daughter pair (assuming 99% isotopic purity), and **(C)** the corresponding corrected compact tandem MS matrix reflecting the labeling of core C-atoms. Correction for natural isotope abundances was performed by 2D-deconvolution (denoted here by “÷”). **(D)** Measured tandem MS matrix for [1,4-^13^C]aspartate *m/z* 418 > 244 parent-daughter pair, as reported by Choi et al. (2012), and **(E)** the corresponding compact tandem MS matrix. **(F)** The corrected compact tandem MS matrix, i.e., reflecting the labeling of only the core C-atoms, was obtained after correction for natural isotope abundances using 2D-deconvolution (denoted here by “÷”).

To illustrate the application of this correction algorithm, we have applied it to correct the tandem MS data that was previously measured for [1,4-^13^C]aspartate. Figure 3D shows the measured tandem MS matrix for the parent-daughter fragment pair *m/z* 418 > 244, as reported by Choi et al. (2012) (Figure 3A). The measured tandem MS matrix was first transformed into the compact tandem MS matrix by row shifting (Figure 3E), and then corrected for natural isotope abundances using 2D-deconvolution, which produced a 3 × 3 matrix that now reflects the labeling of only the core C-atoms of aspartate (Figure 3F). For comparison, we also simulated the theoretical compact tandem MS matrix for [1,4-^13^C]aspartate, assuming 99% isotopic purity of the tracer (Figure 3B), as well as the corresponding corrected compact tandem MS matrix (Figure 3C). Overall, there was very good agreement between the measured and simulated matrices. This example demonstrates that correction for natural isotope abundances can be accomplished easily in a single step by 2D-deconvolution using the compact tandem MS matrices.

## ^13^C-Metabolic Flux Analysis With Tandem MS Data

### EMU Framework for Simulating Tandem MS

**FIGURE 4:**
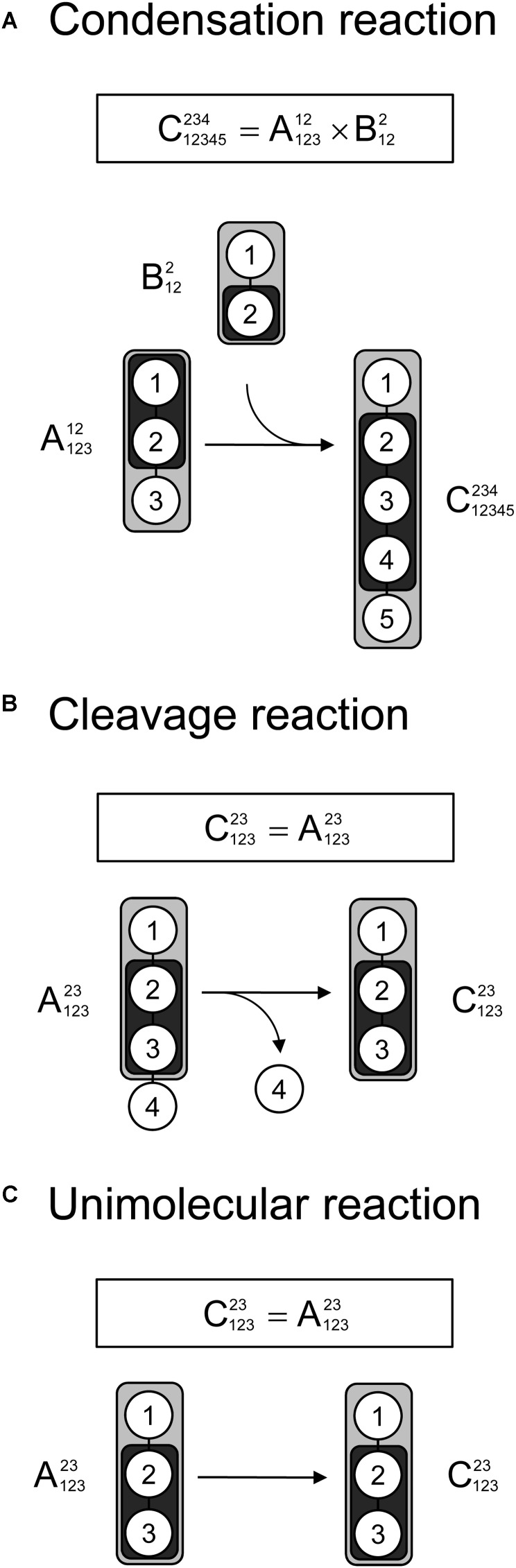
Three types of biochemical reactions **(A)** condensation reaction, **(B)** cleavage reactions, and **(C)** unimolecular reaction, that must be considered for construction of EMU models. The labeling of an EMU product is expressed solely as a function of the labeling of the EMU educts. For the condensation reaction, the compact tandem MS matrix of the EMU product is calculated by 2D-convolution (denoted by “×”) of the compact tandem MS matrices of the EMU educts.

Lastly, we demonstrate that tandem MS data can be used for ^13^C-MFA calculations using the widely used EMU framework. As described in the original EMU paper (Antoniewicz et al., 2007b), there are three types of reactions that must be considered when constructing EMU models: a condensation reaction, a cleavage reaction, and a unimolecular reaction. Figure 4 shows that for all three types of reactions the EMU product can be computed from the corresponding EMU educts. To simulate tandem MS data, compact tandem MS matrices can be used as state variables (note that MID vectors were used as state variables for simulating MS data in the original EMU paper). For an EMU condensation reaction, the compact tandem MS matrix of the EMU product is computed by a 2D-convolution as described above (Figure 4A). For the cleavage reaction and the unimolecular reaction, the compact tandem MS matrix of the EMU product is equal to the compact tandem MS matrix of the EMU educt.

### EMU Decomposition of an Example Metabolic Network Model

**FIGURE 5:**
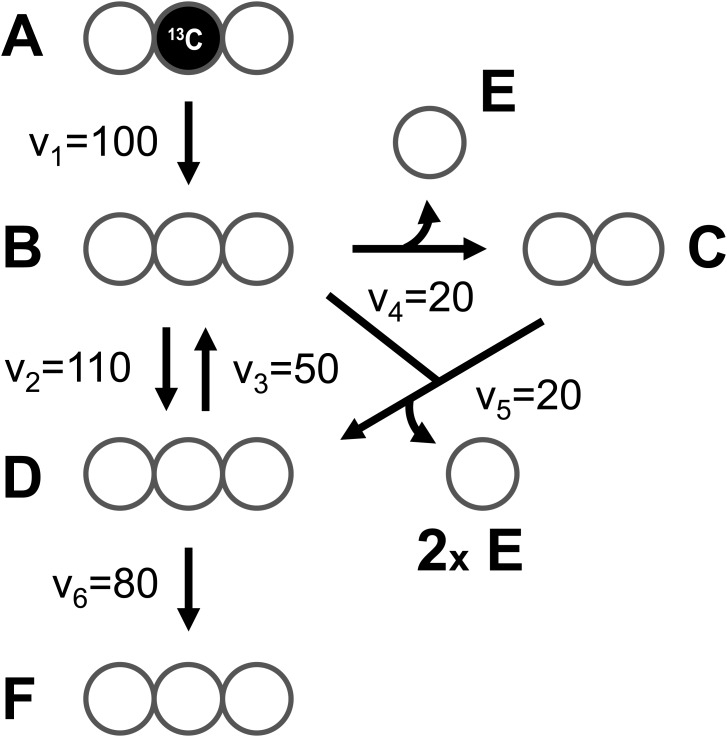
A simple example network model (taken from the original EMU paper), used here to illustrate simulation of tandem MS data using the EMU approach.

At the core of the EMU methodology is the decomposition of the biochemical reaction network into EMU networks, which are then solved subsequently. The EMU decomposition is performed by tracing the origin of carbon atoms of a particular metabolite to carbon atoms of substrates. For more details, the reader is referred to the original EMU paper (Antoniewicz et al., 2007b). In the original EMU framework, EMU decomposition was accomplished by keeping track of C-atoms for the parent fragment only. To generate EMU networks for simulation of tandem MS data, we must also keep track of C-atoms of the daughter fragment. To illustrate this, we will use a simple example network model shown in Figure 5 (that was also used in the original EMU paper), with the corresponding atoms transitions shown in Table 1. The network model was decomposed here in order to simulate tandem MS data for the parent-daughter fragment pair *F*_123_ > *F*_23_. The complete EMU decomposition is shown in Table 2. It is noted that the approach described above is conceptually the same as the tandemers approach described by Tepper and Shlomi (2015).

**Table 1:** Stoichiometry and atom transitions for the reactions in the example metabolic network.

Reaction No.	Reaction stoichiometry	Atom transitions^∗^
1	A → B	abc → abc
2	B ↔ D	abc ↔ abc
3	B → C + E	abc → bc + a
4	B + C → D + E + E	abc + de → bcd + a + e
5	D → F	abc → abc


^∗^For each metabolite atoms are identified using lower case letters to represent successive atoms in the metabolite.

**Table 2:** EMU decomposition for 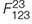 in the example metabolic network.

Reaction No.	EMU reaction
6	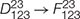
2	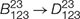
5	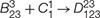
1	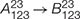
3	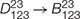
1	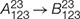
3	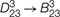
2	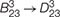
5	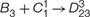
4	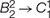
1	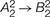
3	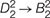
2	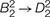
5	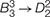
1	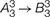
3	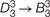
2	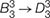
5	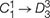
4	B_2_ →C_1_
1	A_2_ →B_2_
3	D_2_ →B_2_
2	B_2_ →D_2_
5	B_3_ →D_2_
1	A_3_ →B_3_
3	D_3_ →B_3_
2	B_3_ →D_3_
5	C_1_ →D_3_


### ^13^C-MFA With Tandem MS Data and the EMU Framework

To determine fluxes with ^13^C-MFA, isotopic labeling must be simulated by solving the EMU network models, which are represented mathematically in the form (see the original EMU paper for more details) (Antoniewicz et al., 2007b):

$$\text{A}{\;}^{*}\;\text{X}=\text{B}{\;}^{*}\;\text{Y}$$

**FIGURE 6:**
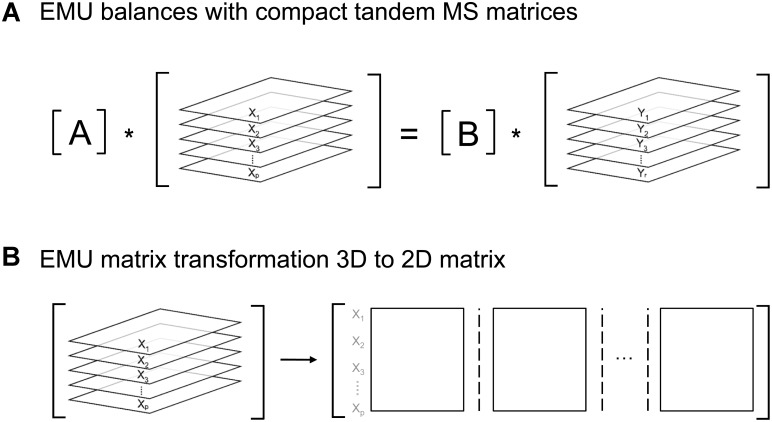
**(A)** Structure of EMU equations for simulation of tandem MS data. **(B)** 3D-matrices X and Y can be transformed into corresponding 2D-matrices for convenience.

Here, A and B are matrices that are functions of fluxes, and X and Y are matrices that contain the unknown and known EMU variables, respectively. In the original EMU framework, each row in the X and Y matrices contained MID vectors of the respective EMU variables. For simulations of tandem MS data, X and Y are now 3-dimensional matrices that contain the compact tandem MS matrices of the respective EMU variables (Figure 6A). For convenience, these 3D matrices can be transformed into 2D matrices as shown in Figure 6B. By performing this 3D-to-2D transformation, the same EMU algorithms can be used to simulate tandem MS data that are used currently to simulate MS data. Thus, this allows current software packages to be upgraded easily to accommodate tandem MS measurements for flux calculations.

**FIGURE 7:**
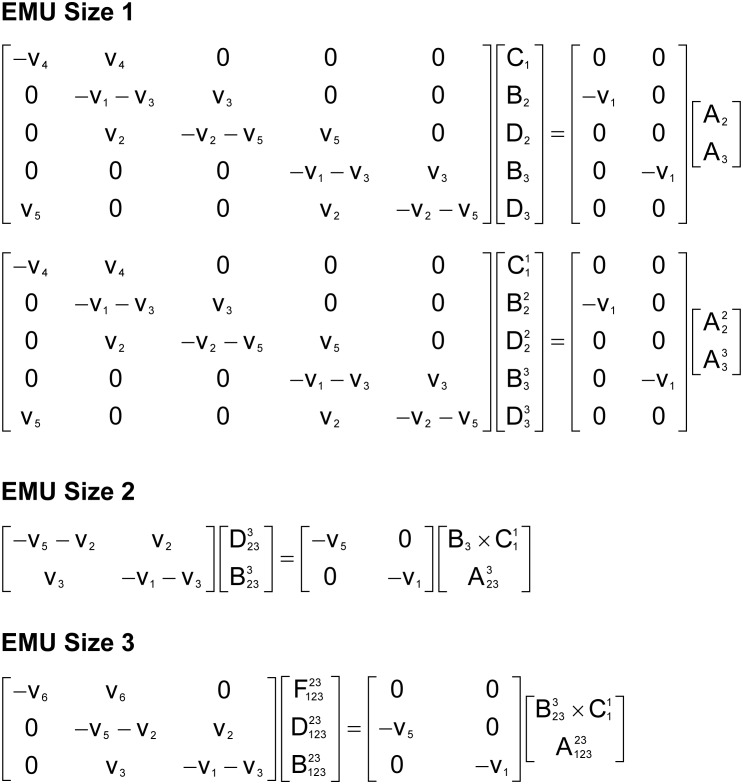
EMU balances for simulation of tandem MS data in the simple example network model (shown in Figure 5). The EMU balances were constructed based on the EMU model decomposition shown in Table 2.

**FIGURE 8:**
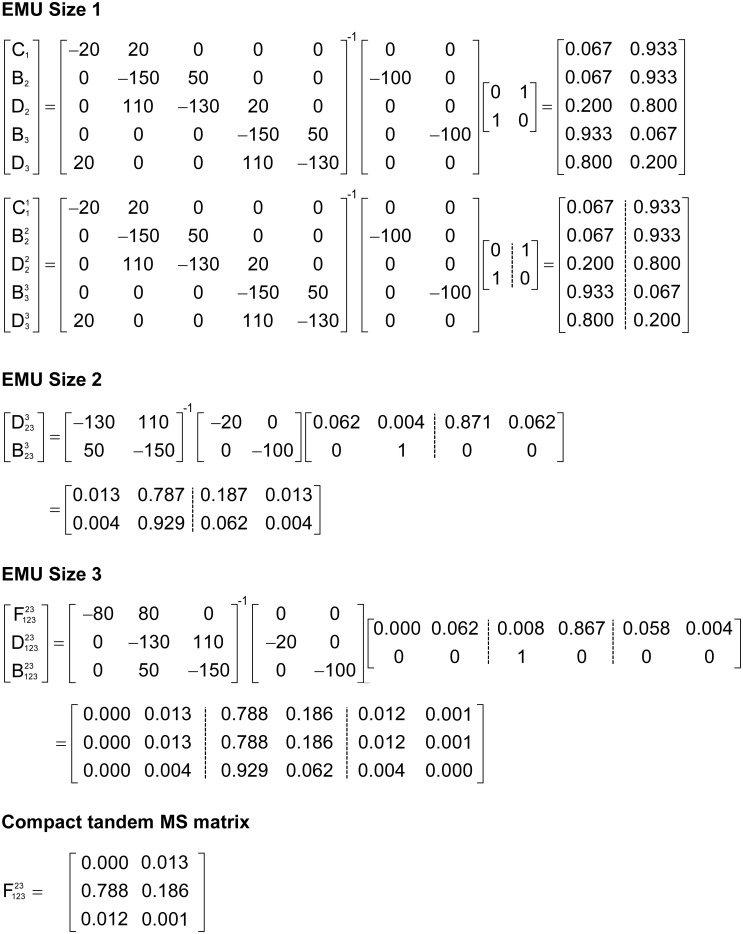
Numerical solution of the EMU balances for the simple example network to simulate tandem MS data for metabolite F (parent fragment C1–C3, daughter fragment C2–C3). For this simulation, the fluxes shown in Figure 5 were used and metabolite A was assumed to be 100% ^13^C-labeled at the second carbon position.

To illustrate the simulation of tandem MS data using the EMU framework and compact tandem MS matrices, Figure 7 shows the EMU balances for the simple example model, and Figure 8 shows the numerical simulation results. Here, we used the fluxes shown in Figure 5 with metabolite A being 100% ^13^C-labeled at the second carbon position. This simple example clearly illustrates that tandem MS data can be efficiently simulated using the existing EMU framework without any major modifications. Recent work has demonstrated that EMU decompositions of large-scale models are computationally tractable (Gopalakrishnan and Maranas, 2015). Thus, the methods and algorithms presented in this paper can be applied to realistically sized network models.

## Concluding Remarks

Tandem mass spectrometry is a promising new analytical approach that provides additional labeling information for ^13^C-flux studies. Previously, we demonstrated that this additional labeling information can significantly improve flux precision and resolution in complex biological systems (Choi and Antoniewicz, 2011). In this paper, we have presented a set of tools and algorithms for efficient simulation of tandem MS data using the EMU framework and for correction of tandem MS data for natural isotope abundances. By building upon the EMU framework, which is used by all current software packages for ^13^C-MFA, we hope to accelerate the acceptance of tandem MS technique by ^13^C-MFA community and encourage software developers to include capabilities for tandem MS ^13^C-flux analysis in future software updates.

## Author Contributions

All authors listed have made a substantial, direct and intellectual contribution to the work, and approved it for publication.

## Conflict of Interest Statement

The authors declare that the research was conducted in the absence of any commercial or financial relationships that could be construed as a potential conflict of interest.
